# Study of Wetland Soils of the Salar de Atacama with Different Azonal Vegetative Formations Reveals Changes in the Microbiota Associated with Hygrophile Plant Type on the Soil Surface

**DOI:** 10.1128/spectrum.00533-22

**Published:** 2022-09-19

**Authors:** Ignacio Ramos-Tapia, Reynaldo Nuñez, Carlos Salinas, Pamela Salinas, Jorge Soto, Manuel Paneque

**Affiliations:** a Laboratory of Metagenomics, Bionostra Chile Research Foundation, San Miguel, Santiago, Chile; b Laboratory of Bioenergy and Environmental Biotechnology, Department of Environmental Sciences and Natural Resources, Faculty of Agricultural Sciences, University of Chile, La Pintana, Santiago, Chile; University of Minnesota

**Keywords:** hygrophile, hyperaridity, hypersalinity, metataxonomics, Salar de Atacama, soil microbiota

## Abstract

Salar de Atacama is located approximately 55 km south of San Pedro de Atacama in the Antofagasta region, Chile. The high UV irradiation and salt concentration and extreme drought make Salar de Atacama an ideal site to search for novel soil microorganisms with unique properties. Here, we used a metataxonomic approach (16S rRNA V3-V4) to identify and characterize the soil microbiota associated with different surface azonal vegetation formations, including strict hygrophiles (Baccharis juncea, Juncus balticus, and Schoenoplectus americanus), transitional hygrophiles (Distichlis spicata, Lycium humile, and Tessaria absinthioides), and their various combinations. We detected compositional differences among the soil surface microbiota associated with each plant formation in the sampling area. There were changes in soil microbial phylogenetic diversity from the strict to the transitional hygrophiles. Moreover, we found alterations in the abundance of bacterial phyla and genera. *Halobacteriota* and *Actinobacteriota* might have facilitated water uptake by the transitional hygrophiles. Our findings helped to elucidate the microbiota of Salar de Atacama and associate them with the strict and transitional hygrophiles indigenous to the region. These findings could be highly relevant to future research on the symbiotic relationships between microbiota and salt-tolerant plants in the face of climate change-induced desertification.

**IMPORTANCE** The study of the composition and diversity of the wetland soil microbiota associated with hygrophilous plants in a desert ecosystem of the high Puna in northern Chile makes it an ideal approach to search for novel extremophilic microorganisms with unique properties. These microorganisms are adapted to survive in ecological niches, such as those with high UV irradiation, extreme drought, and high salt concentration; they can be applied in various fields, such as biotechnology and astrobiology, and industries, including the pharmaceutical, food, agricultural, biofuel, cosmetic, and textile industries. These microorganisms can also be used for ecological conservation and restoration. Extreme ecosystems are a unique biological resource and biodiversity hot spots that play a crucial role in maintaining environmental sustainability. The findings could be highly relevant to future research on the symbiotic relationships between microbiota and extreme-environment-tolerant plants in the face of climate change-induced desertification.

## INTRODUCTION

Salt flats are saline hydrogeological systems frequently associated with arid and hyperarid climates. In these regions, the water table is several centimeters to decimeters below the soil surface (1, 2), which restricts living conditions, requiring adaptations and providing unique biodiversity of high scientific value (3–5). Salar de Atacama forms a part of the Atacama Desert in Chile, and it is the most arid and ancient desert on Earth (6). In this extremely arid and saline zone, the high Andean Tilopozo wetland system is located at the southern edge of the Salar de Atacama basin in the Antofagasta region.

Wetlands are vital ecosystems with high biodiversity. Studies continue to discover new species (7) and new approaches to identify microorganisms that reside in them (8, 9). They are also sources of fiber and food and culturally support local communities (10–12). However, wetlands are also highly vulnerable to climate change. The United Nations Intergovernmental Panel on Climate Change has classified the Andean wetlands as vulnerable (13). The living organisms therein are constantly exposed to different types of abiotic stress, such as hyperosmosis, drought, and ion toxicity (14, 15). Despite the extreme environmental conditions characteristic of Tilopozo, various plant species inhabit and thrive in the area. In contrast, these conditions would be harmful or fatal to nonadapted or foreign plant species.

Tilopozo saline wetlands depend mainly on groundwater discharge from the Monturaqui-Negrillar-Tilopozo (MNT) aquifer. This water supply supports the development of azonal vegetation (10, 16), which is differentiated into strict and transitional hygrophilous plant species (hygrophiles). The roots of the former make direct contact with groundwater, and the representative species of this plant type include Baccharis juncea, Juncus balticus, and Schoenoplectus americanus. The roots of the latter do not make direct contact with water. Rather, these plants absorb moisture via capillary action. The representative species of this plant type include Distichlis spicata, Lycium humile, and Tessaria absinthioides (10). Little is known regarding the microbiota indigenous to the wetlands (meadows, swamps, and wet grasslands) of the arid zones in the central region of the high Andes (12).

Microorganisms play an important role in the regulation of ecological function of wetlands and are critical for maintaining the health of the global ecosystem. Symbiotic microorganisms are crucial for the survival of plants in these extreme ecosystems (17) as they facilitate water and nutrient uptake by the host plant by forming associations with root systems that in turn provide photosynthate (carbon sources) for the growth of microbes. In this manner, a stable survival niche is established and maintained (18–20). Rhizobacteria are associated with host plant roots (15, 20–22) and promote host plant growth directly and indirectly (19, 22).

Studies on this type of microbial community have determined that certain environmental factors, such as aridity, are negatively correlated with rhizobacterial diversity and abundance (23). Wet and dry seasons as well as host plant species are associated with different microbial communities (24, 25). The relative abundance of certain microbial taxa depends on host specificity (26) and soil physicochemistry (24). Hence, soil microorganisms and their host plants mutually support each other and ensure their survival and growth in extreme environments, such as those in Tilopozo.

Due to the marked vulnerability of high Andean wetlands to be affected by climate change in the near future and given the limited studies on the microbiota in Tilopozo, more studies are necessary to understand the diversity of this particular site. In association with the vegetation present in the wetlands, there is a need to know and analyze the composition of the microbial communities (bacteria and archaea) present in the Tilopozo saline wetland system. In this study, the soil microbiota associated with hygrophilous-type plants is profiled: the objective is to characterize and establish the differences in the soil microbiota. We hypothesize that the composition of the microbiota presents differences in the different types of soil and is modulated by the water limitation of the soil. The present study is the first to investigate the composition and diversity of the soil microbiota associated with classes of hygrophiles indigenous to Chile.

## RESULTS

**FIG 1:**
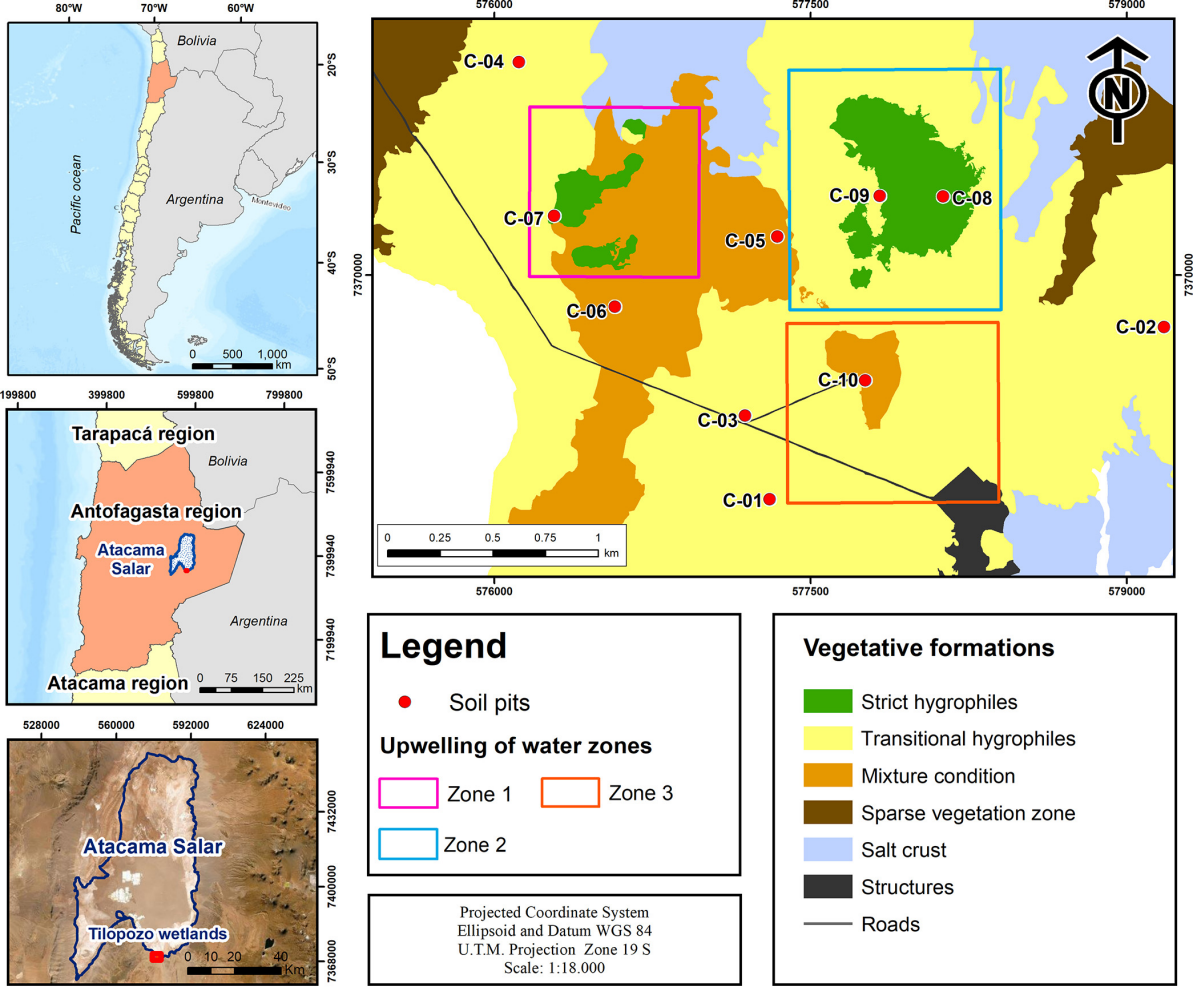
Locations of soil sample excavation sites (pits). Plant formation zones in the Tilopozo study site are also indicated.

In the present study, we characterized the microbiota in the soil samples associated with plant formations in Tilopozo, Salar de Atacama, Antofagasta, Chile (Table 1). In Fig. 1, soil pits are marked as red dots, whereas the plant formation zones are labeled green, yellow, and orange, corresponding to strict, transitional, and mixed hygrophiles, respectively. Microbial DNA from 25 soil samples was subjected to filtration and quality control, yielding 281,654 sequences (average, 11,266; median, 5,754). Taxonomic assignments were made to the microbial kingdom (100%), phylum (99.96%), class (96.95%), order (89.91%), family (87.45%), and genus (79.71%) levels.

**TABLE 1:** Geographical coordinates and vegetation associated with the soil pits excavated in the Tilopozo wetlands^*a*^

Soil pits	Coordinates of UTM WGS-84 (HUSO 19S)		Vegetable formation(s)	Observations
	East (m)	North (m)		
C-01	577,306	7,368,936	Scrub of Tessaria absinthioides	Presence of thick and resistant saline crusts on the surface, dominated by transitional hygrophiles
C-02	579,178	7,369,753		Thick and resistant saline crusts on the surface, evidence of eventual surface runoff, dominated by transitional hygrophiles

C-03	577,189	7,369,332	Grassland of Distichlis spicata	Soil with presence of saline crusts on the surface, dominated by transitional hygrophiles
C-04	576,117	7,371,009		Presence of salts on the surface, surface runoff features and presence of clays, dominated by transitional hygrophiles

C-05	577,343	7,370,182	Grassland of Juncus balticus and Lycium humile	Soils with presence of accumulations of salt on the surface, with presence of sulfur, mixture condition
C-06	576,571	7,369,849		Presence of thick and resistant salt crusts, mixture condition

C-07	576,283	7,370,280	Grassland of Schoenoplectus americanus	Accumulation of salts on the surface, presence of weaker crusts than the others, fully saturated soils, dominated by strict hygrophiles
C-08	578,129	7,370,372		Presence of salt accumulation on the surface and presence of crusts, dominated by strict hygrophiles

C-09	577,829	7,370,375	Grassland with Schoenoplectus americanus and Baccharis juncea	Soil with high organic content, possible presence of peat, on fire at time of visit (recurrent practice by local inhabitants), dominated by strict hygrophiles
C-10	577,759	7,369,500		Soil with high organic content in different stages of decomposition, possible presence of organic horizon, dominated by strict hygrophiles

a Brief descriptions of the environments associated with the soil pits are provided.

**FIG 2:**
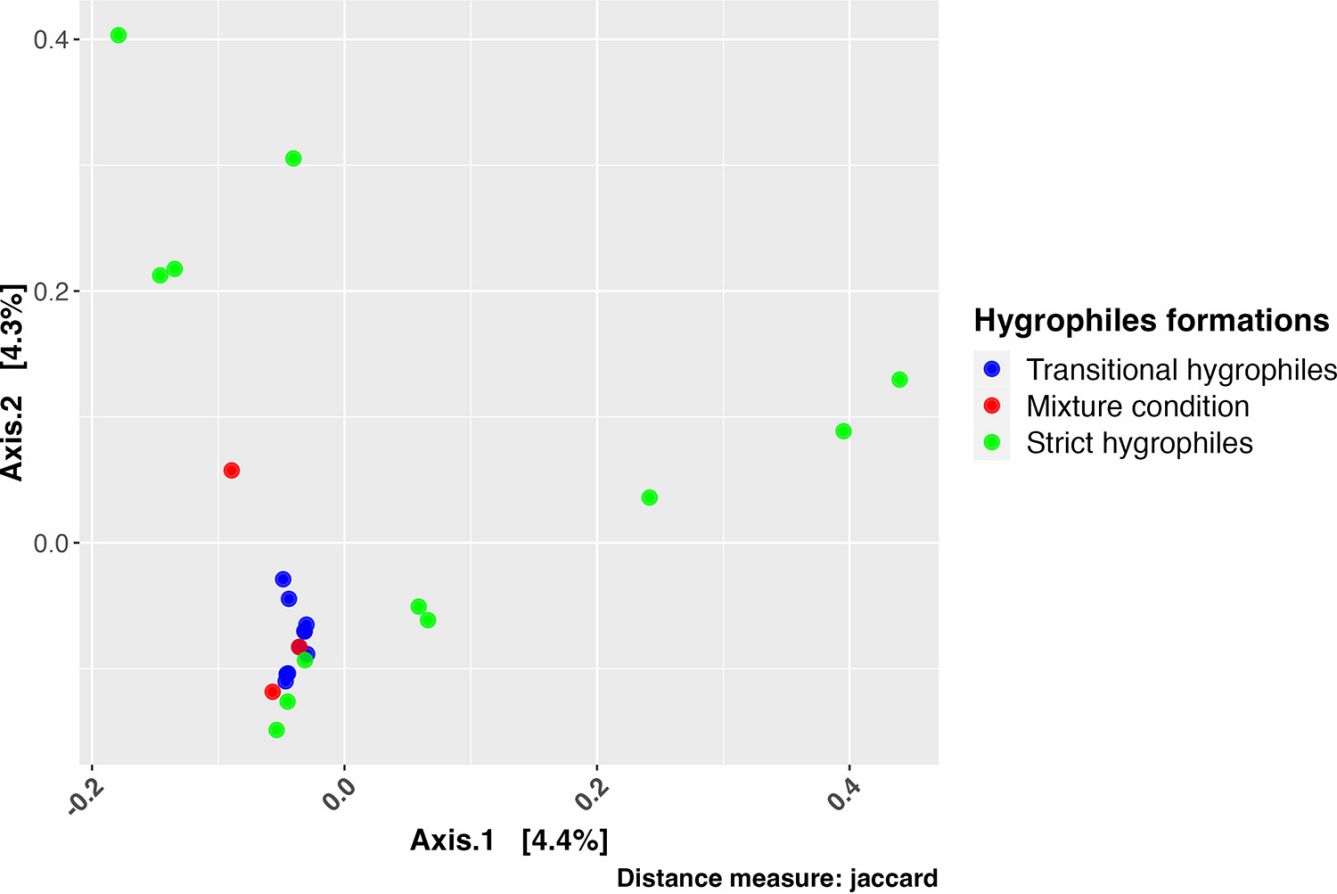
Principal-coordinate analysis plot of samples by hygrophile type. Blue, red, and green correspond to transitional, mixed, and strict hygrophiles, respectively. By permutational multivariate analysis of variance (Adonis) test, Pr (>*F*) = 0.0118 (10,000 permutations).

**FIG 3:**
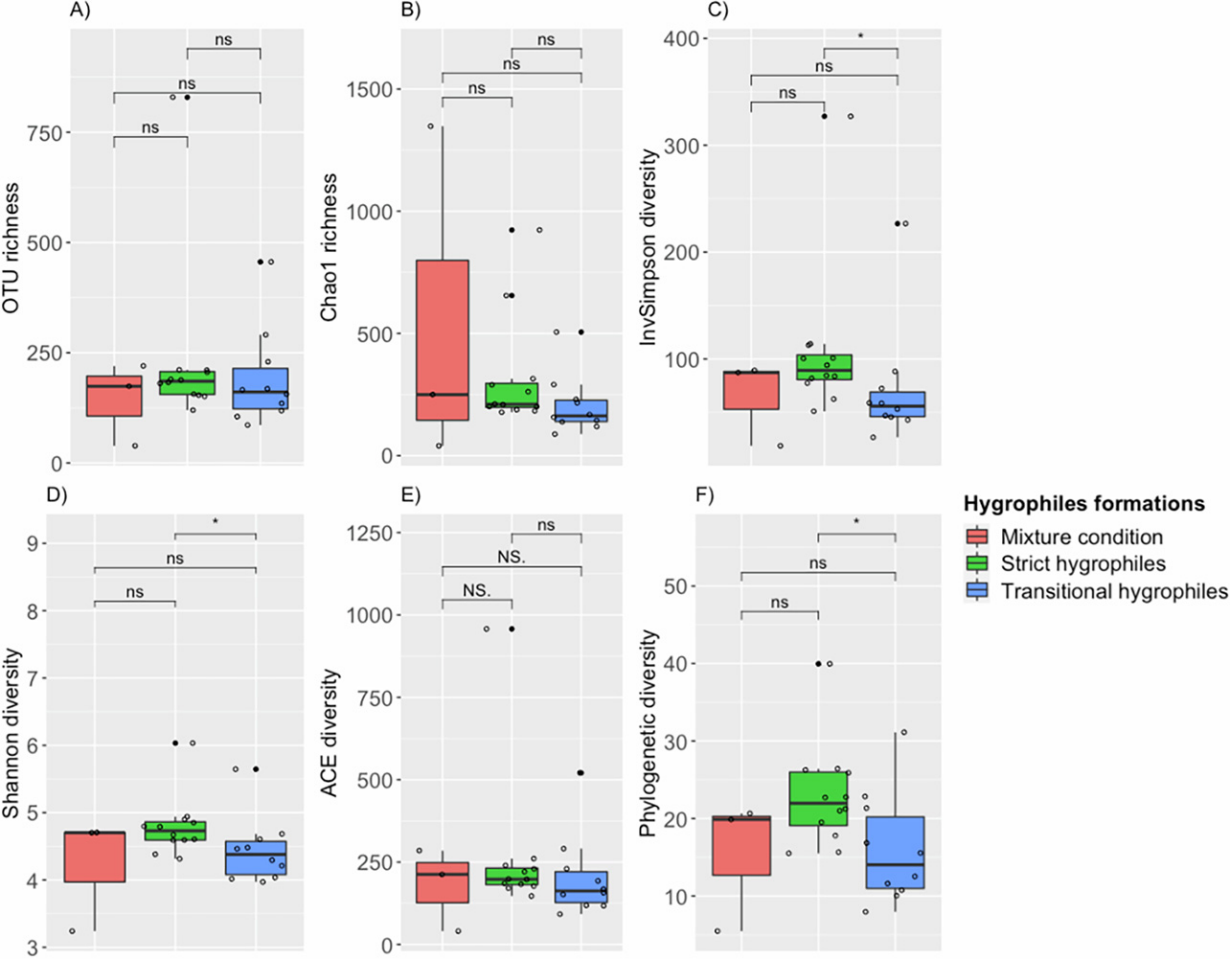
Box plots of α-diversity indices for soil microbiota associated with strict (green), mixed (red), and transitional (blue) hygrophiles. Shown are OTU richness (A) and InvSimpson (B), ACE (C), Chao1 (D), Shannon (E), and phylogenetic (F) diversity (Wilcoxon test, *P* < 0.05). Statistical significance is indicated as follows: NS, not significant at *P* = 1; ns, not significant at *P* > 0.05; *, *P* ≤ 0.05; **, *P* ≤ 0.01; ***, *P* ≤ 0.001; ****, *P* ≤ 0.0001.

For the evaluated microbiota, we used metrics to reveal the differences among the hygrophile formations (strict, transitional, and mixed). The β-diversity analysis revealed significant differences between the strict and transitional hygrophile formations in terms of their Jaccard (permutational multivariate analysis of variance [PERMANOVA], *P* = 0.0118) (Fig. 2), Bray-Curtis (PERMANOVA, *P* = 0.0112), and UniFrac (PERMANOVA, *P* = 0.0002) distances (Fig. S1 and Table S1). We compared α-diversity indices, where pairwise comparisons were made among all formation types. The α-diversity analysis showed significant differences between the strict and transitional hygrophiles (Table S2). InvSimpson diversity was, on average, 67% higher for strict hygrophiles than for transitional hygrophiles, Shannon diversity was, on average, 7% higher for strict hygrophiles than for transitional hygrophiles, and phylogenetic diversity (PD) was, on average, 70% higher for strict hygrophiles than for transitional hygrophiles (Wilcoxon test; *P* < 0.05) (Fig. 3). Hence, values of microbial composition and diversity differed between the strict and transitional hygrophiles.

**FIG 4:**
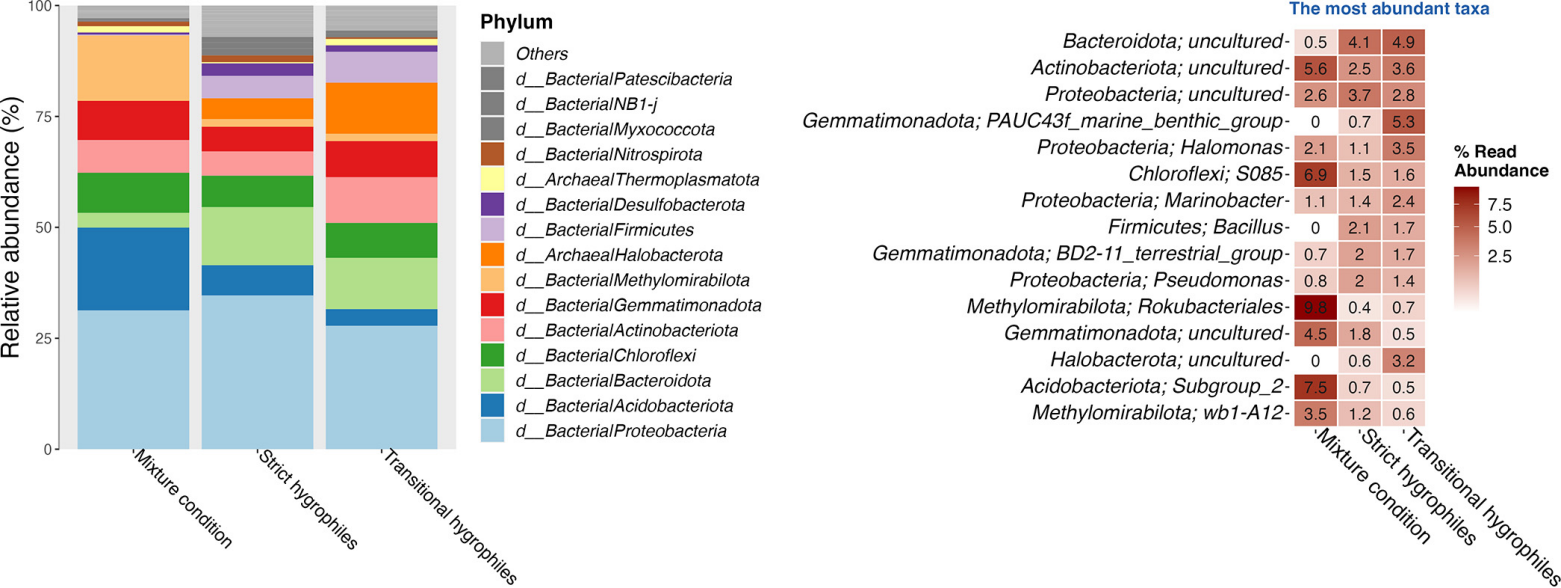
Composition of microbiota associated with hygrophiles. Bar graph (left panel) shows the 15 most abundant bacterial phyla and their respective relative abundance. A heat map (right panel) shows the 15 most abundant genera and their respective phyla.

**FIG 5:**
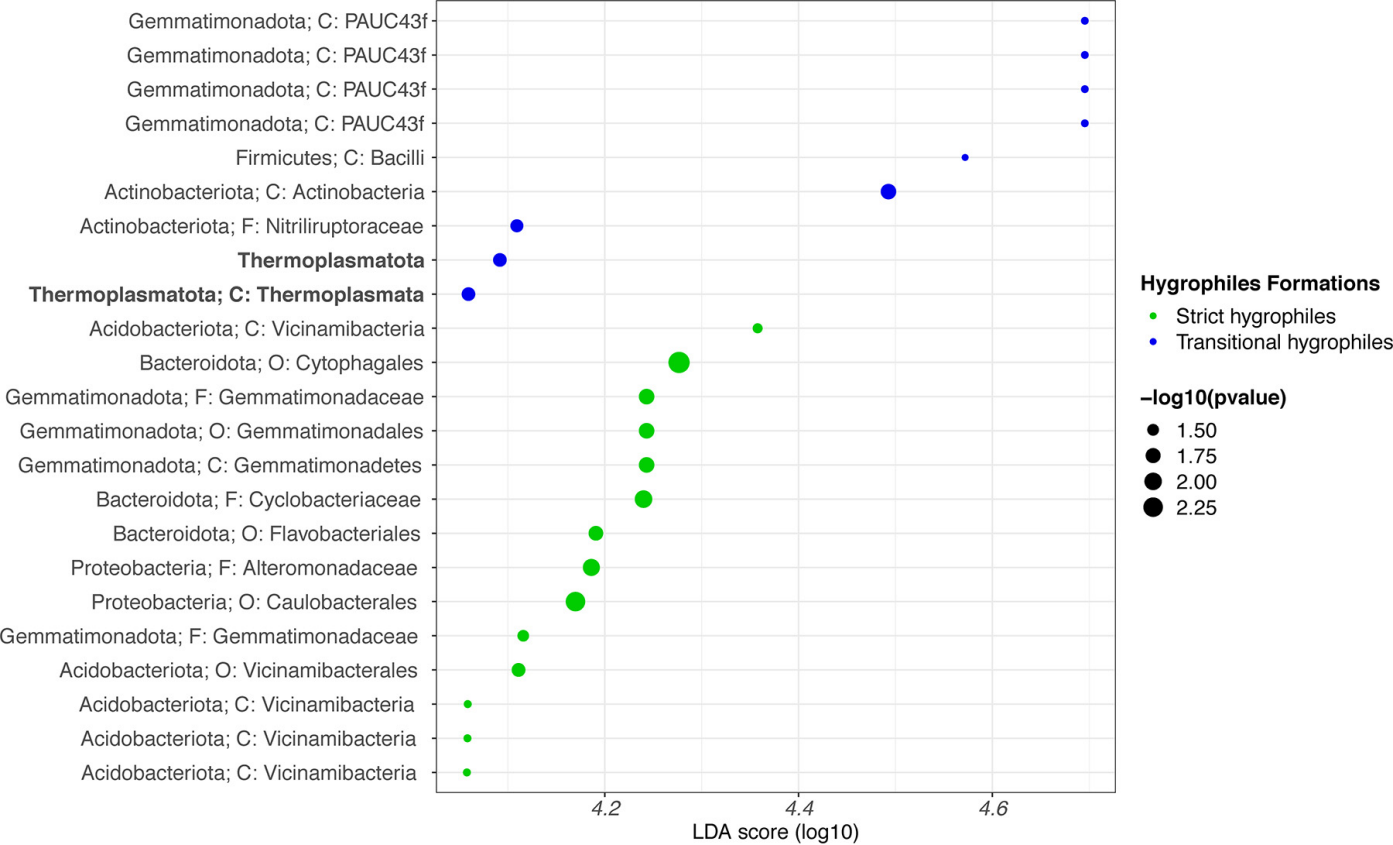
Linear discriminant effect size (LEfSe) microbiota analysis comparing microbiota associated with strict versus transitional azonal hygrophiles. Blue represents transitional hygrophiles, and green represents strict azonal hygrophiles. The panel shows the LDA score of respective taxa of each taxon enriched (Wilcoxon test, *P* < 0.01) and the size of dot represents the −log_10_ *P* value. The taxonomic assignment is up to the lowest possible rank, and taxa in bold correspond to *Archaea*.

The DNA sequences of the soil microbiota revealed 12 dominant phyla on average (>1%), namely, *Proteobacteria* (31.5%), *Bacteroidota* (11.3%), *Actinobacteriota* (7.6%), *Chloroflexi* (7.6%), *Acidobacteriota* (7%), *Gemmatimonadota* (7%), *Halobacteriota* (6.9%), *Firmicutes* (5.2%), *Methylomirabilota* (3.3%), *Desulfobacteriota* (2%), *Myxococcota* (1.1%), and *Nitrospirota* (1%). The most abundant genera comprised uncultured bacteria belonging to the phyla *Bacteriota*, *Proteobacteria*, *Actinobacteria*, *Halobacteriota*, and *Gemmatimonadota*, and included PAUC43f, *Halomonas*, *Bacillus*, *Marinobacter*, BD2-11, Pseudomonas, S085, SAR202, *Saccharospirillum*, and wb1-A12 (Fig. 4). The linear discriminant analysis (LDA) effect size (LEfSe) analysis identified biomarkers distinguishing the microbiota associated with the strict and transitional hygrophiles. *Themoplasmatota* (linear discriminant analysis [LDA] = 4.071; Kruskal-Wallis, *P* < 0.05) and *Gemmatimonadota* (LDA = 4.66; Kruskal-Wallis, *P* < 0.05) were associated with the transitional hygrophiles, whereas *Acidobacteriota* (LDA = 4.32; Kruskal-Wallis, *P* < 0.05), *Bacteroidota* (LDA = 4.24; Kruskal-Wallis, *P* < 0.05), *Proteobacteria* (LDA = 4.03; Kruskal-Wallis, *P* < 0.05), and others (Kruskal-Wallis, *P* < 0.05) were associated with the strict hygrophiles (Fig. 5 and Table S2).

**FIG 6:**
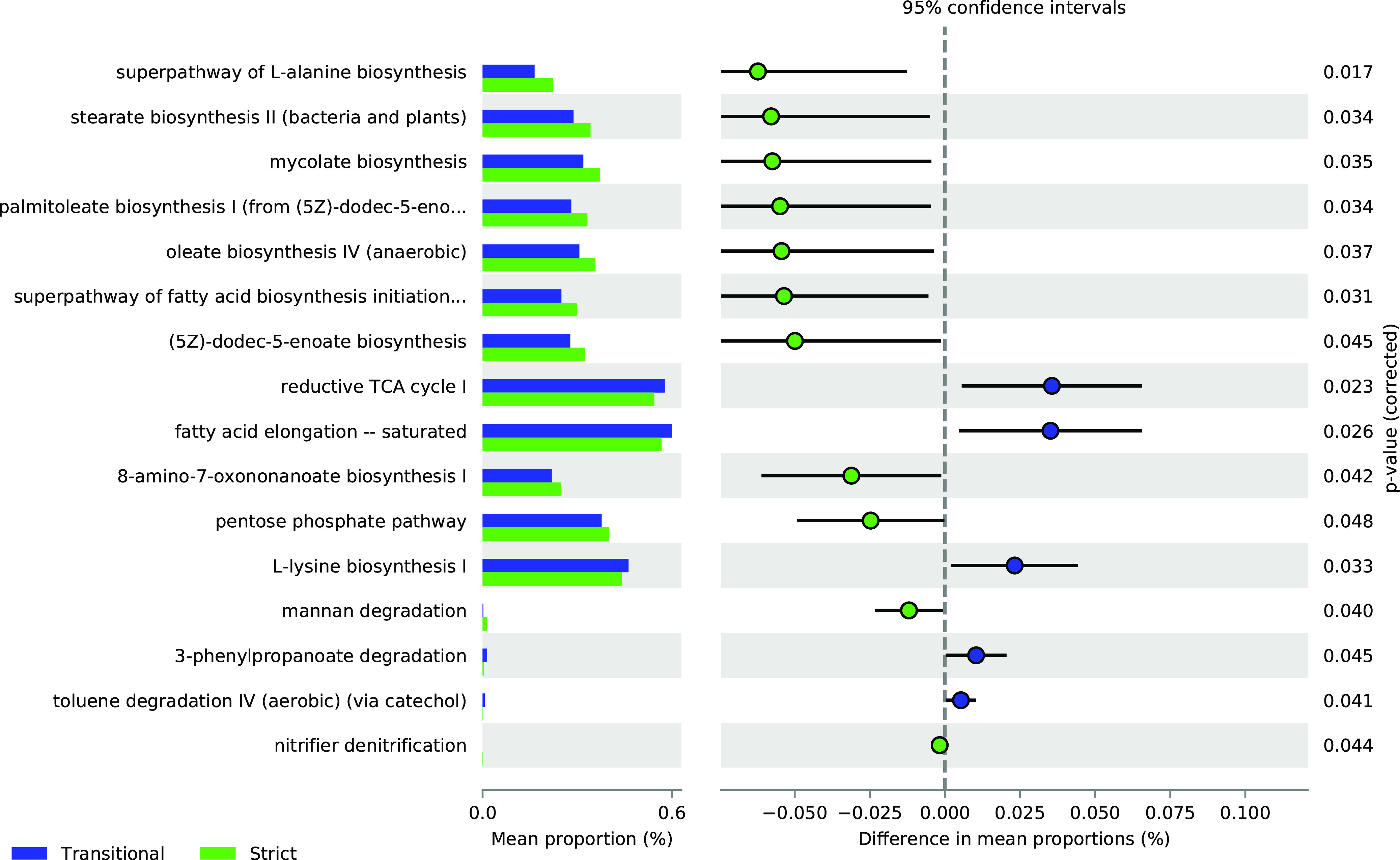
Pathways different between transitional and strict samples. PICRUSt2 was used to infer the abundance of metabolic pathways, and STAMP software was used for visualization and statistical analysis (Welch’s *t* test, CI = 0.95).

We used PICRUSt2 to infer metabolic pathways from the metataxonomic data, which revealed certain differences between strict and transitional hygrophiles. Twelve of these were overrepresented for the strict hygrophiles, namely, the superpathway of l-alanine biosynthesis, stearate biosynthesis II, mycolate biosynthesis, palmitoleate biosynthesis, oleate biosynthesis IV, superpathway initiation of fatty acid (5*Z*-dodec-5-enoate) biosynthesis, 8-amino-7-oxonanoate biosynthesis I, pentose phosphate pathway (PPP), mannan degradation, and nitrifier denitrification. Five of these were overrepresented for the transitional hygrophiles, namely, reductive tricarboxylic acid cycle I, fatty acid elongation, l-lysine biosynthesis, 3-phenylpropanoate degradation, and aerobic toluene degradation IV via catechol (Welch’s *t* test; *P* < 0.05) (Fig. 6).

## DISCUSSION

In the present study, we analyzed the microbial profiles of soil samples collected from a complex and important ecosystem. We detected significant differences in the microbial compositions of the soils associated with the strict and transitional hygrophiles of Tilopozo, Salar de Atacama, Antofagasta, Chile.

### Relative differences between strict and transitional hygrophiles in terms of α and β diversity of the microbiota associated with them.

The soil moisture content influences soil microbial composition and diversity. Drought alters the composition of the fungal and bacterial communities (27–29) in the root microbiome and the rhizosphere (30, 31). An *in vitro* experiment showed drying and rewetting of the soil substantially changed the OTU diversity, indicating mortality, and compositionally altered the relative abundance of *Acidobacteria*, *Alphaproteobacteria*, *Bacteroidetes*, and *Firmicutes* (32). The foregoing findings suggest that substantial differences are expected between soils associated with strict and transitional hygrophiles in terms of their microbial diversity.

As for α diversity, we observed significant differences between strict and transitional hygrophile-associated soils in terms of InvSimpson, Shannon, and phylogenetic diversity indices (*P* < 0.05). This finding indicates that there is great microbial diversity (Shannon index) in soils with strict hygrophile formation (33). In addition, the InvSimpson index showed that soils with strict hygrophile formations are dominated by certain taxa (34, 35) and a greater number of different microorganisms (taxonomically) (25, 36, 37). This may be because soils with water restriction have microorganisms adapted to this abiotic stress, limiting their diversity. The greater diversity may also be due to the higher content of organic matter, at different degrees of decomposition, that can be found in strict hygrophile soils (38). It may also be attributed to the lower salinity (39) of strict hygrophile soils than that of transitional hygrophile soils.

In addition to this, we establish that the composition of the microbiota is different between soils associated with strict and transitional hygrophiles. The Jaccard (40) and Bray-Curtis (41) indices reflect differences in the presence and absence as well as in the abundance of microorganisms; they are unrelated or phylogenetically distant microorganisms (42, 43).

### Comparative soil microbiota compositions between the study region and other study regions.

Studies on soils in Salar de Atacama and Atacama Deserts have reported similar microbial compositions. Both regions have the same phylum composition as the soil of Tilopozo but relatively higher abundance of *Actinobacteria*, *Acidobacteria*, and, to a less degree, *Proteobacteria* (44, 45).

The microbiota compositions of the soil samples from Tilopozo resembled those of the sediment samples from Salar del Huasco (46). Castro-Severyn et al. (46) reported sediment samples with >70% salinity. High salt concentrations in the soils of Tilopozo have also been reported (10). Thus, the observed high soil salt concentrations in these study regions could account for the abundance of certain microbial phyla observed there compared with that in soils from other areas.

In other arid environments (47), the most predominant phyla are *Actinobacteria*, *Acidobacteria*, *Proteobacteria*, and *Bacteroidetes*. In Tilopozo samples, the most predominant phyla are *Proteobacteria*, *Acidobacteria*, *Bacteroidota*, and *Chloroflexi*; this composition is similar to our soil composition if we consider the samples with temperatures between −20 and 19°C (47). However, if the parameters are related to the average annual precipitation, the Tilopozo samples are more similar to the samples in arid zones with precipitations greater than 100 mm/year (47). These results are consistent with the climate of the Tilopozo wetland and the presence of groundwater, which serves as a water resource for strictly hygrophilous plants (10). In contrast, studies conducted in Antarctic soil, with drastically different climate and conditions, have reported the most abundant phylum to be *Actinobacteriota*, followed by *Acidobacteriota*, *Chloroflexota*, and *Proteobacteria* (48). The phyla recorded in the present study in the soils of Tilipozo provide evidence of the selective pressure of certain microorganisms adapted to desert physicochemical conditions as observed in Antarctica and the Atacama Desert (48, 49).

Other places on earth with hot and arid climate are mangrove forests with temperatures up to 37°C and precipitation up to 200 mm in certain months (50, 51). In this extreme environment, the microbiota is modulated and highly specialized by abiotic factors, such as sea level rise, overexploitation, drought, and increased/decreased salinity (52–54). In the mangrove forests, the microbiota composition is dominated by members of phyla *Proteobacteria* (*Alpha*-, *Delta*-, and *Gammaproteobacteria*), *Bacteroidetes*, *Chloroflexi*, and *Cyanobacteria* (51). In contrast, our soil samples have higher relative abundances in *Halobacterota* (*Archaea*), *Methylomirabilota*, and *Firmicutes*. Perhaps the presence of these phyla is due to other factors, such as elevation (3,500 to >5,000 m above sea level) and soils with high levels of UV radiation, the presence of strong oxidizing conditions, and extreme aridity (55). Members of these phyla, especially *Archaea* members, have previously been found in water samples from the Atacama Desert and La Punta-La Brava, a high-altitude lake system (5, 56); therefore, their presence in sectors with a great influence of water has already been reported.

A microbiota that does not show significant differences between strict and transitional hygrophiles is the microbiota of the mixed zone (strict and transitional). These soil samples are reported to be dominated by member of the phylum *Methylomirabilota*, the candidate phylum of anaerobic methanotrophs (57), involved in the process of methane oxidation with electron acceptors (58). Perhaps for a biotechnological application, isolation of bacteria such as “*Candidatus* Methylomirabilis oxyfera” (59) would be of great interest.

The change in microbiota composition in the mixed hygrophile zone is not surprising. Other studies have shown how the soil microbiome response differed significantly based on the vegetation coverage, such as pine forest (60), evergreen broad-leaf forest, coniferous forest, subalpine dwarf shrubs, alpine meadows (61), and different native plants in desert areas (62).

### Biomarker microorganisms in strict and transitional hygrophile soil samples.

We used LEfSe (63) to detect biomarker microorganisms in strict and transitional hygrophile soils. *Thermoplasmatota* are highly abundant marine planktonic *Archaea* (64). This new phylum was recently proposed after a metagenomic-assembled genome (MAG) analysis of seawater samples collected from the coast of Sydney, Australia (65). *Thermoplasmatota* members constitute 76% of this type of taxon in surface seawater samples (66). However, *Thermoplasmatota* members have also been found in peatlands and included methanogenic archaea affecting atmospheric CH_4_ emissions (67). Future research, such as comprehensive genome analysis (68), co-occurrence network analysis (51, 69, 70), and assembly of the particular *Thermoplasmatota* genome using MAGs (65, 71) with whole metagenomic sequencing, could clarify the contributions of *Thermoplasmatota* to the rhizospheres of hygrophiles in Tilopozo.

The second particular and poorly studied phylum associated with both types of hygrophiles is *Gemmatimonadota*. This phylum was initially identified using massive sequencing based on the sequences of the 16S rRNA gene (72) reported in different environments, such as sediments (73–75), permafrost (76–78), rhizosphere (79–81), and soil (82, 83). Currently there are six cultivable species, and two of them are capable of anoxygenic photosynthesis (84, 85); this ability to adapt is perhaps related to obtaining energy in sites with high selective pressure. In addition, recent studies have found that this phylum along with others in the soil and rhizosphere is related to the bioremediation or reduction of certain metals, such as cadmium (86, 87). Specific taxa, including *Gemmatimonadota*, are associated with growth, disease prevention ability, and resistance to cadmium when primarily colonizing the rhizosphere (86). Further studies, focusing on rhizospheric soil, will be required to determine if these qualities of the phylum determine the survival of plants in this hostile environment of the Atacama Desert.

Strict hygrophiles are principally associated with *Acidobacteriota*, *Bacteroidota*, and *Proteobacteria*. In ecological and biological terms, it is not surprising that a soil without water restriction presents a variety of microorganisms (88). However, a study focusing on fungi (internal transcribed spacer [ITS]) could provide more information on the diversity and soil pressure associated with strict hygrophiles as these appear to be more sensitive indicators of soil water content than bacteria (89). Soils associated with strict hygrophiles have a greater variety of associated microorganisms as the selective pressure is lower than that in soils with transition hygrophiles.

### Impact of enrichment metabolic pathways on the soils of strict and transitional hygrophiles.

Elucidation of the functions of bacterial communities can provide important insights into the ecosystem processes and the mechanisms to which these communities contribute. Here, we used PICRUSt2 (90) and inferred substantial differences between the soils of the strict and transitional hygrophiles in terms of the predominant metabolic pathways of their microbiota.

In both cases, we detected pathways related to the biosynthesis of various amino acids and lipids. Nevertheless, their sources differed. The naturally occurring compound 3-phenylpropionate is generated from the degradation of organic matter, aromatic compounds, and lignins (91). This pathway is characteristic of the microbiota in the soils of transitional hygrophiles. It is indicative of extreme microbial halophiles, such as *Halofex* spp., that can use aromatic compounds as their sole energy and carbon sources (92). Certain microbiota in the soils of transitional hygrophiles can degrade toluene and substituted benzoic acids into Krebs cycle intermediates (91). In turn, the latter may be used as energy sources by Pseudomonas spp. and others (93). Other microorganisms indigenous to the soil of Tilopozo could also use these metabolic routes to obtain energy under adverse conditions, such as drought, hypersalinity, and diurnal temperature extremes. Hyperarid environments generally have nitrogen limitation (94); therefore, microorganisms play an important role in this task. Recent studies have shown that the functional potential of the microbiota in hypersaline and hyperarid environments favors metabolic pathways related to nitrogen metabolism and amino acid synthesis (95, 96). This is observed in soil samples associated with hygrophiles, where the metabolic pathways associated with amino acid biosynthesis have been overrepresented.

Future research is required to determine the importance and contributions of the microorganisms in the rhizospheres of various hygrophiles and establish whether these bacteria and plants are, in fact, symbiotically associated. Subsequent investigations should analyze Tilopozo soil and its entire microbiome. Exploration of the mycobiota of Tilopozo could furnish novel insights into the contributions of fungi to the soil ecosystems there. Soil mycobiota in extreme environments exhibits thermotolerance (97) and adaptive compositional changes in response to warming and drought (98).

### Limitations.

Amplicon sequencing is a useful tool to characterize the microbiota of a particular niche. However, the information is limited only to the composition of the sample. This technique does not take into consideration the possible metabolic functions encoded in the genome of the microorganisms present. Future studies could address this using more complex molecular techniques that, in addition to the analysis of composition of the microbiota, include its functional potential and the genes expressed by the microbiota, using metagenomics and metatranscriptomics, respectively (99).

In addition, an interesting focus for future studies is the application of dual transcriptome sequencing (dual RNA-seq) to understand the metabolic pathways of the rhizospheric microbiota and plants.

### Conservation and biodiversity.

Wetlands are important sources of biodiversity (10, 12), and Tilopozo is a high Andean wetland system with unique characteristics. Areas of these types harbor unique microorganisms that have not yet been discovered, as well as unique associations between the rhizospheric microbiota and plants. These associations could play a key role in the resistance of wetland plants to different abiotic factors that can be used in agriculture as biofertilizers (30, 100).

The greatest measure to maintain this place and continue the study of biodiversity would be the declaration of the status of Chile as a protected area. In this way, the permanence of this ecosystem is ensured; moreover, it could be free of human intervention, and the total conservation of life forms that reside here could be attained.

### Conclusion.

Tilopozo in Salar de Atacama, Antofagasta, Chile, is characterized by arid, hypersaline soils, adjacent saline water bodies, and wetlands. In this extreme environment live strict and transitional hygrophile plants. We found significant differences in the microbiota compositions of the soils associated with each plant type. The observed variations in soil microbial diversity and composition among plant types may be explained by the relative differences in the hydric capacities of the soils associated with them. The rhizospheres of these plant types should nonetheless be analyzed to determine the roles of microorganisms therein. Our results demonstrated that certain microbial phyla are characteristic of each soil type and could, therefore, be used as biomarkers or associate with plants to survive. These findings provide a clue regarding relevant microorganisms in soils associated with drought or water limitations.

*Thermoplasmatota* and *Gemmatimonadota* are interesting phyla that warrant further research, as they may contribute the tolerance and persistence of hygrophilic plants. However, more advanced analytical techniques must be applied to establish their functions in this extreme ecosystem. Future research should endeavor to identify factors that account for the robustness of the microbial communities of this harsh environment. To achieve this, the functions, metabolic profiles, and plant symbiotic associations of these microbial communities should be explored.

## MATERIALS AND METHODS

### Study site.

The research area is the Tilopozo saline wetland system located in the southern sector of Salar de Atacama in the Antofagasta region, Chile (23°46′42′′S, 68°14′39′′W) (10). The elevation of this region is ~2,305 m above sea level (5). Tilopozo is characterized by low levels of precipitation (historical average of 1.67 ± 1.65 mm and maximum of 14.57 ± 16.43 mm) concentrated between February and April. In the dry period between September and November, precipitation is 0.00 mm. The region also presents with high thermal oscillations. The average annual temperature is 16.51 ± 3.31°C, the coldest month is July (minimum temperature of −0.72 ± 1.63°C), and the warmest month is December (maximum temperature of 31.82 ± 1.64°C). The average monthly evapotranspiration is 163.63 mm. Minimum evapotranspiration occurs in the winter months of June and July, whereas maximum evapotranspiration occurs in the summer months of December and January (10).

The soils in Tilopozo are characterized by surface saline encrustations, concretions, and/or accumulations. However, in certain sites, water rises near or at the soil surface and supports the development of strict and transitional hygrophiles (azonal vegetation) (10). The soils in Tilopozo displaying surface salt accumulation have salinity levels ranging from 5.8 dS m^−1^ (moderately saline) to 223.0 dS m^−1^ (extremely saline). The lithology of the regional MNT basin contributes to the high concentrations of Ca^2+^, Mg^2+^, K^+^, Na^+^, and Cl^−^ in the soil.

### Sample collection.

Enriched soil samples were collected from a total of 10 pits and distributed as follows: strict (*n* = 4), transitional (*n* = 4), and mixed hygrophile formations (*n* = 2). In each pit, the horizons of the soil profile were identified, and then samples were collected (4 to 5 per pit). The collection by soil profile involved extraction around the roots (maximum of 3 cm) with a sterile spatula. The soil content was deposited in sterile 50-mL Falcon tubes (Fisher Scientific, Waltham, MA, USA) to three-fourths the volume. The tubes were immediately sealed to avoid environmental contamination, labeled, and stored at 4°C during transport to the laboratory.

### DNA extraction and sequencing.

A previously reported method (101) was applied for DNA extraction and sequencing, but it was modified because of the high salt concentrations in the samples. The soil was processed in two stages, and microbial DNA was isolated from 2 g of each soil sample. In the first stage, bacteria and archaea were isolated from the substrate using a cytoplasmic extraction buffer (CEB) composed of 1% (wt/vol) polyethylene glycol (PEG 8000) and 1 M NaCl in Milli-Q water (EMD Millipore, Billerica, MA, USA). The pH was adjusted to 9.2 with 0.2 N NaOH. Forty-five milliliters of CEB was mixed with 1 g of soil in each 50-mL tube, and the suspensions were gently mixed by inverting the tubes for 3 min. The tubes were then centrifuged at 220 × *g* for 5 min to remove coarse soil particles. The pellets were discarded, and the supernatants were transferred to new tubes and centrifuged at 10,000 × *g* for 20 min. The supernatants were discarded and the pellets comprising the microbial biomass were collected.

In the second stage, the microbial pellets were suspended in 100 μL of sterile water and transferred to the columns provided in the DNeasy Powersoil kit (Qiagen, Hilden, Germany). A homogenizer (Hi Lab, model SK2210HP) was operated at 40 Hz for 5 min to break the cells and release their DNA. The DNA was eluted with 100 μL of Tris buffer and stored at −20°C until further use.

Fifty nanograms of DNA was fixed to DNA-stable columns (Biomatrica, San Diego, CA, USA) and delivered to Genewiz, Inc. (South Plainfield, NJ, USA), for genomic library preparation and 16S rRNA sequencing by paired-end MiSeq (2× 250 bp) (Illumina, San Diego, CA, USA). The genomic DNA (gDNA) libraries were generated using the MetaVx kit (Genewiz, Inc.). The gDNA was used to generate 16S rRNA amplicons spanning the bacterial and archaeal V3 and V4 hypervariable regions. The amplicons were amplified with forward primers (CCTACGGRRBGCASCAGKVRVGAAT) and reverse primers (GGACTACNVGGGTWTCTAATCC) (102–104).

### Microbiota analysis.

The fastqs files supplied by the facilities were processed in QIIME2 (105). The reads were trimmed to 240 bp, and their quality was set and maintained with PHRED 20. The new unified sequences (operational taxonomic units [OTUs]) and MAFFT were used to perform the alignment (106). The maximum likelihood (ML) phylogenetic tree was inferred with FastTree (107).

OTU abundance, taxonomy, phylogeny, and metadata were integrated into a phyloseq object for subsequent analyses with the Phyloseq package v1.38.0 (108). Quality control filters were applied (109). The following were excluded: (i) samples with <1,000 reads, (ii) unassigned OTUs, (iii) samples with the mean number of reads per taxon of >1e−5, and (iv) OTUs not detected more than twice in ≥10% of all samples.

The calculation of α diversity was performed in R with the function estimate_richness (Phyloseq version 1.38.0) for observed, Chao1, ACE, Shannon, Simpson, and InvSimpson indices. Phylogenetic diversity was estimated using Picante package of R (version 1.8.2) (110). Pairwise comparisons are made among all formation types using the Wilcoxon test (*P* < 0.05).

β diversity (Jaccard, Bray-Curtis, and phylogenetic UniFrac weighted and unweighted) dissimilarity among samples was evaluated using principal-coordinate analysis. Each index was compared using permutational multivariate analysis of variance in the “vegan” package v2.5-7 in R (111) with the adonis function directly based on the algorithm of Anderson (63). Significance was determined for 10,000 permutations.

Linear discriminant analysis effect size (LEfSe) was performed on the azonal species factor between the strict and transitional hygrophiles using a high-dimensional biomarker discovery algorithm (112) and Kruskal-Wallis analysis (*P* < 0.05) with the “microbiomeMarker” package (v1.1.2) (113) in R (v4.1.1). All analyses were performed with R (v4.1) and ggplot2 v3.35 (114) in Rstudio (v2021.9.2.382) for visualization. Metabolic pathways were predicted using the OTU sequences and their abundance in PICRUSt2 (v2.4.1) relative to microbial functional signatures (90). Differentially represented pathways were calculated using Welch’s *t* test (115) (confidence interval [CI] = 0.95) and the Benjamini-Hochberg correction false-discovery rate (90) in STAMP (116).

### Data availability.

All raw sequences have been deposited in the National Center for Biotechnology Information (NCBI) database (Bethesda, MD, USA) under BioProject accession no. PRJNA603831.
