# Supplementary material for: Study of Wetland Soils of the Salar de Atacama with Different Azonal Vegetative Formations Reveals Changes in the Microbiota Associated with Hygrophile Plant Type on the Soil Surface
Source: Microbiol Spectr. 2022 Sep 19;10(5):e00533-22. doi: 10.1128/spectrum.00533-22 (PMC9602281; doi:10.1128/spectrum.00533-22)
Supplement: Supplemental file 1 — Fig. S1 and Tables S1 to S3. Download spectrum.00533-22-s0001.pdf, PDF file, 0.2 MB [file spectrum.00533-22-s0001.pdf]

SUPPLEMENTARY FIGURE LEGENDS

Fig. S1. Principal coordinates analysis plot of samples by hygrophile type. Blue, red, and green correspond to transitional, mixed, and strict hygrophiles, respectively. Left panel shows UniFrac diversity (Adonis; P = 0.0002) and the right panel shows Bray–Curtis diversity (Adonis; P = 0.0112). Permutational multivariate analysis of variance with 10,000 permutations.

Table S1. Statistics of β-diversity. PERMANOVA analysis of Jaccard, Bray–Curtis, and phylogenetic UniFrac weighted and unweighted distances.

Table S2. Statistics of α-diversity. Pairwise comparisons of α-diversity indices were made among all formation types. Wilcoxon test; $p < 0.05$.

Under the table

The columns from left to right show the Index to compare, groups to compare (columns 2 and 3), p-value, adjusted p-value, significant p-value, and nomenclature of significance. The following convention of symbols indicates statistical significance: NS: $p = 1$; ns: $p > 0.05$; *: $p \leq 0.05$; **: $p \leq 0.01$; ***: $p \leq 0.001$;****: $p \leq 0.0001$.

Table S3. Statistics of LEfSe. Biomarkers identified and enriched by group.

Under the table

The columns from left to right show the taxa with taxonomic annotation to the lowest level, group in which they are enriched, LDA score, p-value, and adjusted p-value.

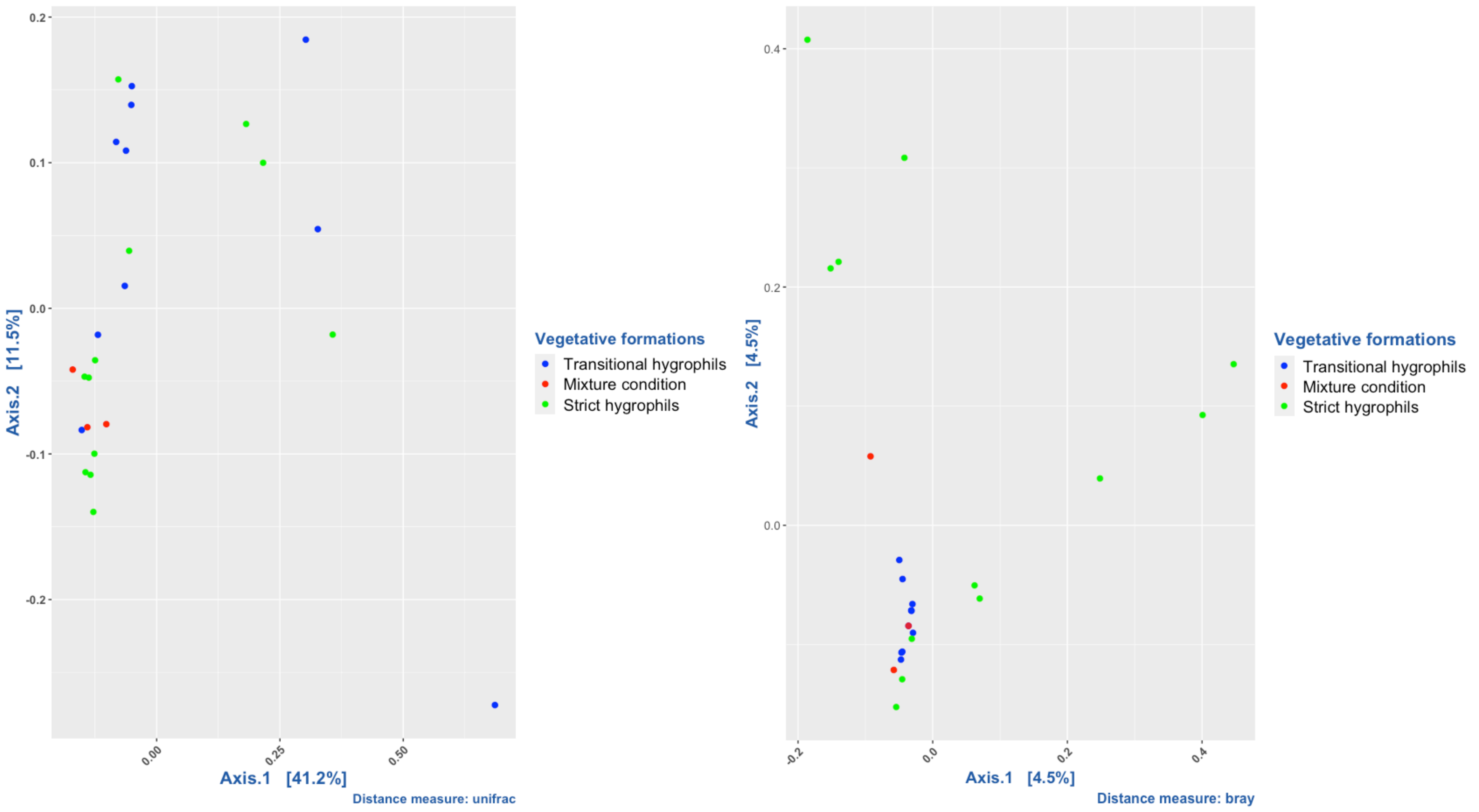
0.2
0.1
0.0
-0.1
-0.2
Axis.2 [11.5%]
0.00
0.25
0.50
Axis.1 [41.2%]
Distance measure: unifrac
Vegetative formations
Transitional hygrophils
Mixture condition
Strict hygrophils
0.4
0.2
0.0
Axis.2 [4.5%]
-0.2
0.0
0.2
0.4
Axis.1 [4.5%]
Distance measure: bray
Vegetative formations
Transitional hygrophils
Mixture condition
Strict hygrophils

Beta index	DF	SumsOfSqs	MeanSqs	F.Model	R2	PR(>F)
Unifrac	2	1.118	0.559	1.746	0.137	0.0002
Wunifrac	2	0.367	0.184	1.702	0.134	0.0736
Bray-curtis	2	10.016	0.508	1.020	0.085	0.0112
Jaccard	2	1.008	0.504	1.010	0.084	0.0118
Permutational multivariate analysis of variance with 10,000 permutations.						

Wilcoxon tests

Index	Group 1	Group 2	p	p.adj	p.signf	Nomenclature of significance
Observed	Strict hygrophils	Transitional hygroph…	0.356	1.000	0.360	ns
Observed	Strict hygrophils	Mixture condition	0.718	1.000	0.720	ns
Observed	Transitional hygrophils	Mixture condition	0.937	1.000	0.940	ns
Chao1	Strict hygrophils	Transitional hygroph…	0.080	0.240	0.080	ns
Chao1	Strict hygrophils	Mixture condition	0.945	1.000	0.950	ns
Chao1	Transitional hygrophils	Mixture condition	0.692	1.000	0.690	ns
InvSimpson	Strict hygrophils	Transitional hygroph…	0.011	0.033	0.011	*
InvSimpson	Strict hygrophils	Mixture condition	0.448	0.900	0.448	ns
InvSimpson	Transitional hygrophils	Mixture condition	0.811	0.900	0.811	ns
Shannon	Strict hygrophils	Transitional hygroph…	0.025	0.075	0.025	*
Shannon	Strict hygrophils	Mixture condition	0.448	0.900	0.448	ns
Shannon	Transitional hygrophils	Mixture condition	0.692	0.900	0.692	ns
ACE	Strict hygrophils	Transitional hygroph…	0.180	0.540	0.180	ns
ACE	Strict hygrophils	Mixture condition	1.000	1.000	1.000	ns
ACE	Transitional hygrophils	Mixture condition	1.000	1.000	1.000	ns
PD	Strict hygrophils	Transitional hygroph…	0.036	0.110	0.036	*
PD	Strict hygrophils	Mixture condition	0.180	0.360	0.180	ns
PD	Transitional hygrophils	Mixture condition	0.937	0.940	0.937	ns

**Feature**	**enrich_group**	**ef_lda**	**pvalue**	**padj**
Gemmatimonadota: C: PAUC43f.	Transitional hygrophils	4.668	0.046	0.046
Gemmatimonadota: C: PAUC43f.	Transitional hygrophils	4.668	0.046	0.046
Gemmatimonadota: C: PAUC43f.	Transitional hygrophils	4.668	0.046	0.046
Gemmatimonadota: C: PAUC43f.	Transitional hygrophils	4.668	0.046	0.046
Firmicutes; C: Bacilli	Transitional hygrophils	4.581	0.049	0.049
Actinobacteriota; C: Actinobacteria	Transitional hygrophils	4.499	0.016	0.016
Acidobacteriota; C: Vicinamibacteria	Strict hygrophils	4.326	0.039	0.039
Bacteroidota; O: Cytophagales	Strict hygrophils	4.241	0.003	0.003
Bacteroidota; F: Cyclobacteriaceae	Strict hygrophils	4.203	0.010	0.010
Gemmatimonadota; C: Gemmatimonadetes	Strict hygrophils	4.190	0.016	0.016
Gemmatimonadota; O: Gemmatimonadales	Strict hygrophils	4.190	0.016	0.016
Gemmatimonadota; F: Gemmatimonadaceae	Strict hygrophils	4.190	0.016	0.016
Proteobacteria: F: Alteromonadaceae	Strict hygrophils	4.163	0.011	0.011
Actinobacteriota; F: Nitriliruptoraceae	Transitional hygrophils	4.124	0.026	0.026
Proteobacteria: O: Caulobacterales	Strict hygrophils	4.123	0.006	0.006
Acidobacteriota: O: Vicinamibacterales	Strict hygrophils	4.096	0.023	0.023
Bacteroidota; O: Flavobacteriales	Strict hygrophils	4.087	0.019	0.019
Gemmatimonadota; F: Gemmatimonadaceae	Strict hygrophils	4.078	0.032	0.032
**Thermoplasmatota**	Transitional hygrophils	4.071	0.023	0.023
**Thermoplasmatota; C: Thermoplasmata**	Transitional hygrophils	4.050	0.023	0.023
Acidobacteriota; C: Vicinamibacteria	Strict hygrophils	4.016	0.046	0.046
Acidobacteriota; C: Vicinamibacteria	Strict hygrophils	4.014	0.046	0.046
Acidobacteriota; C: Vicinamibacteria	Strict hygrophils	4.014	0.046	0.046
